# Calcium-sensing receptor-mediated NLRP3 inflammasome activation in rheumatoid arthritis and autoinflammation

**DOI:** 10.3389/fphys.2022.1078569

**Published:** 2023-01-06

**Authors:** Lina Emilia Werner, Ulf Wagner

**Affiliations:** Rheumatology Unit, Department of Internal Medicine III, University of Leipzig, Leipzig, Germany

**Keywords:** rheumatoid arthritis, calciprotein particle, inflammation, NLRP3 inflammasome, monocytes, calcium-sensing receptor

## Abstract

The calcium-sensing receptor (CaSR) is expressed in many cell types – including immune cells and in particular circulating monocytes. Here, the receptor plays an important physiological role as a regulator of constitutive macropinocytosis. This review article provides an overview of the literature on the role of the calcium sensing receptor in the context of inflammatory processes. Special emphasis is laid upon the importance for monocytes in the context of rheumatoid arthritis. We have shown previously, that stimulation of the receptor by increased extracellular Ca^2+^ ([Ca^2+^]_ex_) triggers a pro-inflammatory response due to NLRP3 inflammasome assembly and interleukin (IL)-1β release. The underlying mechanism includes macropinocytosis of calciprotein particles (CPPs), which are taken up in a [Ca^2+^]_ex_-induced, CaSR dependent manner, and leads to strong IL-1β release. In rheumatoid arthritis (RA), this uptake and the resulting IL-1β release is significantly increased due to increased expression of the receptor. Moreover, increased [Ca^2+^]_ex_-induced CPP uptake and IL-1β release is associated with more active disease, while CaSR overexpression has been reported to be associated with cardiovascular complications of RA. Most importantly, however, in animal experiments with arthritic mice, increased local calcium concentrations are present, which in combination with release of fetuin-A from eroded bone could contribute to formation of CPPs. We propose, that increased [Ca^2+^]_ex_, CPPs and pro-inflammatory cytokines drive a vicious cycle of inflammation and bone destruction which in turn offers new potential therapeutic approaches.

## Introduction

The calcium-sensing receptor (CaSR) is a multifunctional receptor involved in a wide variety of (patho-) physiological processes. It was first identified in bovine parathyroid cells in 1993 (Brown et al., 1993) and has since been found to be functionally important for all vertebrates (Herberger and Loretz, 2013). The receptor is central for the maintenance of systemic calcium homeostasis since it is involved in the regulation of parathyroid hormone, calcitonin, calcitriol, and fibroblast growth factor-23 (FGF23) (Brown, 2013). Accordingly, expression is high in chief cells of the parathyroid glands and thyroid C cells (Garrett et al., 1995), but also in cells of the renal tubules (Riccardi et al., 1995) and in various cell types of the small intestine (Chattopadhyay et al., 1998). The receptor is also involved in bone remodeling as well as bone resorption and is important for osteoclast and osteoblast function (Chang et al., 1999; Cianferotti et al., 2015). In addition to its importance in calcium homeostasis and in calcitropic tissues, the receptor also contributes to the coordination of numerous other cellular mechanisms due to its expression in the cardiovascular and gastrointestinal systems, pancreatic islet cells, and the central nervous system (Hannan et al., 2018). The functionality of the receptor is determined by its polyvalence. In addition to Ca^2+^, other cations (such as Gd^3+^, Ba^2+^, and Mg^2+^) and various amino acids (for example poly-L-arginine, amyloid-β-peptide, polyamines) serve as ligands (Conigrave et al., 2000; Zhang et al., 2016a; Geng et al., 2016). As recently summarized by Gorkhali and colleagues, the receptor is involved in many other processes such as cell proliferation, cell differentiation, cytoskeletal rearrangements, regulation of certain ion channels (Gorkhali et al., 2021), but also in neurotransmission (Lo Giudice et al., 2019) and nutrient sensing (Liu et al., 2018).

In recent years, numerous studies have examined the importance of CaSR in various pathological contexts (Vahe et al., 2017; Leach et al., 2020; Sundararaman and van der Vorst, 2021; Tőke et al., 2021). Consistent with its systemic distribution and functionality, genetic or acquired CaSR-mediated perturbations are pathophysiological relevant for calcium homeostasis, but also for non-calcitropic tissues (Hannan et al., 2018). Loss-of-function mutations are associate with hypercalcemia, while gain-of-function mutations mediate hypocalcemia (Hendy et al., 2000). In addition, several polymorphisms have been described that predispose to various diseases not exclusively related to mineral metabolism or that affect the response to certain therapeutic modalities such as calcimimetic treatment (Rothe et al., 2005; März et al., 2007; Tőke et al., 2021). A non-calcitropic disease associated with polymorphisms of CaSR is, for example, breast cancer (Campos-Verdes et al., 2018). Furthermore, autoimmune reactions mediated by the formation of antibodies against the extracellular domain of the receptor have been described to cause hypocalciuric hypercalcemia (Kemp et al., 2014; Weetman, 2015; Vahe et al., 2017). Alzheimer’s disease (Chiarini et al., 2016), epilepsy (Kapoor et al., 2008) and ischemic brain injury have also been associated with dysfunctions or dysregulations of the receptor (Hannan et al., 2018), and it also plays a role in the context of vascular complications such as myocardial ischemia, vascular calcification, hypertension, obesity and atherosclerosis (Sundararaman and van der Vorst, 2021).

The involvement of the receptor in diverse pathological processes highlights its cellular profound, pleiotropic importance. The purpose of this review is to highlight the role of the receptor in an inflammatory and immunological context. Specifically, the involvement of CaSR in the inflammatory pathogenesis of rheumatoid arthritis (RA) will be presented and discussed below.

## Signaling pathways induced by calcium-sensing receptor

The CaSR is active as a homodimer or heterodimer localized in the plasma membrane (Gama et al., 2001; Chang et al., 2007) and belongs to class C of G-protein-coupled receptors (GPCRs) (Nørskov-Lauritsen and Bräuner-Osborne, 2015; Møller et al., 2017). Accordingly, the receptor features seven transmembrane domains that are connected *via* intracellular and extracellular loops, enabling signal transduction from the extracellular space to intracellular second messengers (Rosenbaum et al., 2009). Ligand binding occurs *via* a large N-terminal extracellular domain, which features the structure of a bivalved Venus flytrap (Zhang et al., 2016b; Geng et al., 2016). The intracellular domain enables heterogeneous signal transduction and mediates the interaction with various G-protein subunits, in particular: G_q/11_, G_i/o,_ and G_12/13_ (Conigrave and Ward, 2013; Hannan et al., 2018). It has become clear that the activation state of GPCRs, including CaSR, is multidimensional. Crucially, different ligands can modulate the transduction of signals and shape their intracellular consequences (Thomsen et al., 2012). This signaling, termed biased signaling, is caused by the association of different ligands with distinct conformational states of the receptor, which in turn influence the induction of downstream signaling pathways (Leach et al., 2014). The corresponding activation of the various G-proteins, but also the affinity of the CaSR for different agonists seems to depend on the cell type (Huang and Miller, 2007). In general, the G-protein-mediated signal transduction is associated with complex intracellular signaling pathways, which result in particular in a modulation of gene transcription (Gorvin, 2018a).

The important G_q/11_-mediated signal transduction results in the activation of the key effector protein, phospholipase C (PLC). This leads to hydrolysis of the important cellular second messengers inositol-1,4,5-trisphosphat (IP3) and diacylglycerol (DAG) (Brown et al., 1993; Chang et al., 1998). IP3 induces the intracellular release of Ca^2+^ from cellular stores by activating corresponding receptors in the endoplasmic reticulum (ER) (Conigrave and Ward, 2013). DAG activates protein kinase C, which is involved in the regulation of various signal transductions, such as the activation of mitogen-activated protein kinases (MAPKs). MAPK signaling is also induced by the release of intracellular Ca^2+^ and is particularly involved in the regulation of transcription *via* the induction of p38 kinase, JUN N-terminal kinase and extracellular-signal regulated kinase 1 and 2 (Gorvin, 2018a; Hannan et al., 2018). Another major signaling cascade is induced by activation of G_i/o_. This induces the activation of adenylate cyclase (AC) and suppresses the production of cyclic adenosine monophosphate (cAMP) (Chang et al., 1998; Kifor et al., 2001). Upon the concomitant inhibition of protein kinase A, this provides another activation pathway for MAPK signaling (Ward, 2004; Gorvin, 2018a). Activation of the CaSR has also been described to mediate MAPK-signal transduction *via* a mechanism involving β-arrestin proteins, independent of G-protein activation (Thomsen et al., 2012; Gorvin et al., 2018b).

## Calcium-sensing receptor in immune cells and inflammation

Several studies have shown that the receptor itself exerts a modulatory effect on the immune response. For example, CaSR has been found to be involved in inflammatory processes relevant to allergic asthma (Yarova et al., 2015). In airway smooth muscle cells, activation of CaSR can be induced by inflammatory cationic proteins and increased [Ca^2+^]_ex_, which correlate with asthma severity. In these studies, activation of the receptor was observed in association with intracellular Ca^2+^ mobilization, which was accompanied by a decrease in intracellular cAMP and induction of MAPK (Yarova et al., 2015). CaSR is also relevant in pathophysiological processes of white adipose tissue in obesity. It was observed that in visceral adipose tissue, stimulation of CaSR causes increased expression of pro-inflammatory cytokines (Cifuentes et al., 2012). The importance of the receptor in hypertension has also been investigated. Increased expression of the receptor was found to be associated with aortic fibrosis in spontaneously hypertensive rats and linked to pro-inflammatory responses (Zhang X et al., 2019). Another study in mice showed that in the brain, where the receptor is expressed by neurons, microglia, and astrocytes, subarachnoid hemorrhage-induced CaSR activation leads to a decrease in neurological function and thus promotes neuronal degeneration (Wang et al., 2020). This study suggests that the serine/threonine kinase CaMKII, which is involved in the modulation of various cellular functions as a sensor of intracellular [Ca^2+^], is involved in this pro-inflammatory CaSR-mediated process (Wang et al., 2020). In general, selective inhibition of the receptor with NPS 2143 has been shown to inhibit inflammatory processes (Mine and Zhang, 2015; Yarova et al., 2015; Zhang X et al., 2019; Wang et al., 2020). Mouse models have clearly illustrated that pretreatment with NPS 2143 inhibits the migration of inflammatory cells and thus the production of pro-inflammatory cytokines in lipopolysaccharide (LPS)-induced acute lung injury (Lee et al., 2017).

Immune cell-induced pro-inflammatory processes mediated by CaSR activity have been studied in various pathological contexts (see Table 1) (Liu et al., 2021). Overall, three different CaSR-mediated functions have been described in immune cells: Induction of cytokine secretion, cell migration and macropinocytosis. Accordingly, the focus has been on T lymphocytes (Li et al., 2013; Wu C et al., 2015; Wu Q Y et al., 2015), neutrophils (Zhai et al., 2017; Chang et al., 2018) and especially monocytes/macrophages (Yamaguchi et al., 1998b; Yamaguchi et al., 1998a; Olszak et al., 2000; Xi et al., 2010; Rossol et al., 2012b; Malecki et al., 2013; Paccou et al., 2013; Liu et al., 2015; Canton et al., 2016; Séjourné et al., 2017; D'Espessailles et al., 2020; Jäger et al., 2020).

**TABLE 1:** 

CaSR in immune cells and inflammation.			
Cell type cell system	Function	Related intracellular signaling pathways	Pathological relevance
**T lymphocytes**	Cytokine release (Li et al., 2013)	NF-κB pathway, MAPK (Li et al., 2013; Wu C. et al., 2015; Zeng et al., 2016)	• Sepsis (Wu C et al., 2015; Wu Q. Y. et al., 2015)
• Primary CD4^+^ T cells (Chang et al., 2018)	Migration (Chang et al., 2018)	PI3-Cdc42 cascade,	• Acute myocardial infarction (Zeng et al., 2016; Zeng et al., 2018)
• Human/rat peripheral blood T lymphocytes (Li et al., 2013; Wu C et al., 2015; Wu Q. Y. et al., 2015; Zeng et al., 2016)		Ca^2+^/calmodulin dependent myosin phosphorylation (Chang et al., 2018)	• Immune response, inflammation (Chang et al., 2018)
**Neutrophils**	Cytokine release (Zhai et al., 2017)	NF-κB pathway (Zhai et al., 2017)	• Acute myocardial infarction (Ren et al., 2020)
• Human/rat peripheral blood polymorph nuclear neutrophils (Zhai et al., 2017; Ren et al., 2020)		NLRP3 *via* PLC-IP3 pathway, ER-Ca^2+^ release (Ren et al., 2020)	• Immune response, inflammation (Chang et al., 2018)
• Neutrophil-like HL-60 (Chang et al., 2018)	Migration (Chang et al., 2018)	PI3-Cdc42 cascade,	
		Ca^2+^/calmodulin dependent myosin phosphorylation (Chang et al., 2018)	
**Monocytes Macrophages**	Cytokine release (Yamaguchi et al., 1998a)	NLRP3 *via* PLC-IP3 pathway (Rossol et al., 2012b)	• RA (Rossol et al., 2012b; Jäger et al., 2020)
• Human/mouse peripheral blood monocytes/macrophage (Rossol et al., 2012b; Lee et al., 2012; Canton et al., 2016; Jäger et al., 2020)		NLRP3 *via* PLC-IP3 and decreased cellular cyclic AMP (Lee et al., 2012)	• Myocardial infarction (Liu et al., 2015)• Peripheral artery disease (Malecki et al., 2013)• CAPS (Lee et al., 2012)
• mouse monocyte/macrophage cell line (J774) (Yamaguchi et al., 1998a)	Migration (Olszak et al., 2000; Boudot et al., 2010)	Akt, PLC (Boudot et al., 2010) PI3-Cdc42 cascade,	• Obesity (Thrum et al., 2022)• Osteoarthritis (Séjourné et al., 2017)• Atherosclerosis (Malecki et al., 2013)
• THP-1 macrophages (Xi et al., 2010; D'Espessailles et al., 2020)		Ca^2+^/calmodulin dependent myosin phosphorylation (Chang et al., 2018)	• Orchitis (Su et al., 2020)
• U937 monocytes (Chang et al., 2018)	Macropinocytosis (Canton et al., 2016; Jäger et al., 2020)	PLC induced accumulation of lipid mediators, activation of guanine nucleotide exchange factors, branching of actin network (Canton et al., 2016)	• Immune response, inflammation (Chang et al., 2018)

Extended and modified according to (Liu et al., 2021).

Regarding CaSR-induced cytokine release, for example, it became clear that activation of the receptor on T lymphocytes leads to the secretion of pro-inflammatory cytokines such as IL-6 and tumor necrosis factor (TNF) (Li et al., 2013). In sepsis as a systemic inflammatory response syndrome, activation of the CaSR can induce apoptosis of T lymphocytes through the signaling pathway *via* PLC-IP3 (Wu C et al., 2015). In addition, it has been demonstrated that CaSR mediates pro-inflammatory responses in T lymphocytes (Zeng et al., 2016) but also in neutrophils could be important in acute myocardial infarction (Ren et al., 2020). Previously, it was shown that induction of CaSR-dependent pro-inflammatory responses by M1 macrophages also appear to be relevant in the presence of this cardiac complication (Liu et al., 2015).

In this inflammatory context, it is also of relevance that the promoter of *CaSR* is regulated by pro-inflammatory cytokines (Hendy and Canaff, 2016). This implies the potential for a feedback loop that fuels inflammation (Kelly et al., 2011; Fetahu et al., 2014; Séjourné et al., 2017). For example, Canaff and colleagues demonstrated that IL-6 induces increased gene transcription of *CaSR* in the kidney, thyroid, and parathyroid glands (Canaff et al., 2008). Moreover, pro-inflammatory cytokines have been reported to increase expression of the receptor in preadipocytes, adipocytes, and the LS14 adipocyte line of human visceral adipose tissue (Cifuentes et al., 2010). In general, receptor-related inflammation may be the cause, but also a consequence, of a local disturbance in calcium homeostasis (Li et al., 2013).

Elevated calcium concentrations at sites of acute or chronic inflammation or infection may further stimulate CaSR-mediated chemotaxis, particularly of monocytes (Olszak et al., 2000). For example, using a mouse model, Olszak and colleagues showed that subcutaneous injection of 5 mM calcium chloride induces marked infiltration of monocytes. Staining of monocytes on corresponding skin sections clarified the effect of calcium as a chemoattractant (Olszak et al., 2000). This mechanism is significant for the migration of monocytes circulating in the bloodstream and the initialization and modulation of the innate immune response (Olszak et al., 2000).

Yet another CaSR-mediated function is of particular relevance for monocytes and macrophages. Canton and colleagues identified the CaSR as a mediator of induction and maintenance of constitutive macropinocytosis. This extends the understanding of the receptor, which was previously only known as a chemotactic guide to the site of inflammation, to the realization that the receptor is also essential for antigen presentation (Canton et al., 2016). Monocytes/macrophages are professional phagocytes which continuously internalize their environment to screen for foreign and harmful markers. The uptake of extracellular components occurs *via* actin-mediated invaginations of the membrane (Bohdanowicz et al., 2013). Canton and colleagues showed, that CaSR signaling induces the accumulation of phosphatidic acid and phosphatidylinositol (3,4,5)-trisphosphate (PIP₃) at the plasma membrane. These signaling lipids cause the polymerization of actin at the plasma membrane through complex coordination of specific nucleators. Inhibition of the receptor by drugs, but also culture under calcium-free conditions, blocks the formation of PIP₃. This results in inhibition of the Rho-GTPase family members Rac1/Cdc42, which are essential for cytoskeleton reorganization, and thereby inhibits constitutive macropinocytosis (Canton et al., 2016; Canton, 2018).

## NOD-like receptor protein-3 inflammasome

The best studied inflammasome complex is the NOD-like receptor protein-3 (NLRP3) inflammasome. Inflammasomes are multimeric protein complexes assembled from precursor proteins, which have to be available in sufficient concentrations in the cell (Bryant and Fitzgerald, 2009). Inflammasome assembly and activation always require two separate signals, the first of which is triggered in response to various pathogen-associated molecular patterns (PAMP) or damage-associated molecular patterns (DAMP) after Toll-like receptor (TLR) activation (Liston and Masters, 2017). This priming step involves NF-κB-dependent activation of mRNA expression and protein translation (Bauernfeind et al., 2009) of the adaptor molecule ASC (apoptosis-associated speck-like protein containing a CARD), caspase-1, and an inflammasome sensor, NLR protein, which varies between the different types of inflammasomes. In addition, the priming step also leads to deubiquitination of NLRP3 by the Lys-63-specific deubiquitinase BRCC3 (Py et al., 2013), allowing subsequent oligomerization.

The second signal can vary widely between different cell types and different inflammasome types. Activation of the NLRP3 inflammasome begins with oligomerization of de-ubiquitinylated NLRP3. Oligomerized NLRP3 then recruits ASC through pyrin domain-pyrin domain interactions (Lamkanfi and Dixit, 2014), which forms long filaments and assembles into a large protein complex, in which pro-caspase-1 is recruited (Franklin et al., 2014). In the final step of activation, the complex contracts into so-called SPECKs, bringing pro-caspase-1 molecules into close proximity to each other. This starts the process of autoproteolytic maturation and self-activation of pro-caspase-1 into active caspase-1 (Franklin et al., 2014) and subsequent IL-1β maturation begins.

In addition to cytokine cleavage, mature caspase-1 also cleaves gasdermin-D, triggering the formation of a pore-forming protein complex and leading to pyroptosis, a pronounced cell death characterized by swelling, membrane rupture, and release of cell contents, including pro-forms of cytokines such as pro-IL-1β, into the extracellular space (Shi et al., 2017). In the presence of extracellular stains and activated caspase-1, pro-IL-1β released from dying cells can be cleaved after pyroptotic cell death, leading to amplification of the inflammatory signal.

The classical inflammasome-dependent members of the extended IL-1 cytokine family are IL-1β and IL-18, both of which have a caspase-1 cleavage site (Afonina et al., 2015). IL-1α, on the other hand, which is also released under certain conditions following inflammasome activation (Rossol et al., 2012b), matures independently of caspase-1 but is cleaved by various proteases such as elastase, granzyme B, and mast cell chymase.

A pathogenetic role for the NLRP3 inflammasome has been originally shown for gout, periodic fever syndromes, and type II diabetes (Dinarello, 2009). More recently, a contribution of NLRP3 has been shown for a wider range of inflammatory and autoimmune diseases, including atherosclerosis (Duewell et al., 2010; Rajamäki et al., 2010) and myocardial infarction (Toldo and Abbate, 2018), while vitiligo associates with NLRP1 mutations (Jin et al., 2007; Grandemange et al., 2017). Lately, new results have indicated an involvement of NLRP3 activation with obesity (Vandanmagsar et al., 2011), depression (Kaufmann et al., 2017) aging (Youm et al., 2013) and breast cancer (Ershaid et al., 2019), among others, which indicates a potentially far greater role of this inflammasome in the pathogenesis of common diseases. In atherosclerosis, NLRP3 is activated by cholesterols crystals (Duewell et al., 2010; Rajamäki et al., 2010) and oxidized LDL (Jiang et al., 2012; Sheedy et al., 2013; Liu et al., 2014; Oury, 2014).

## Calcium-sensing receptor-mediated NOD-like receptor protein-3 inflammasome activation

In view of the alterations of extracellular calcium concentrations under various pathological conditions, our group investigated the effects of increased [Ca^2+^]_ex_ on peripheral blood monocytes. Concurrent with Lee and colleagues, we were the first to demonstrate that extracellular Ca^2+^ act as damage-associated molecular pattern (DAMP) and trigger activation of the NLRP3 inflammasome (Rossol et al., 2012b; Lee et al., 2012). Both studies independently reported that the activation signal for the assembly of the NLRP3 inflammasome is mediated by CaSR and that accumulation and elevation of intracellular [Ca^2+^] occur in association with activation of the IP3/Ca^2+^ pathway. The study by Lee and colleagues described the relevance of this mechanism for autoinflammation in cryoporin-associated periodic syndromes (CAPS). The results indicates that CaSR-induced inflammasome activation triggers bouts of fever in this autoinflammatory diseases (Lee et al., 2012). Examination of macrophages within this study suggested an additional CaSR-mediated decrease in intracellular cAMP preventing its actual inhibition of inflammasome assembly (Lee et al., 2012). In our experiments with monocytes no significant involvement of a modulation of intracellular cAMP in [Ca^2+^]ex-induced inflammasome activation was detectable (Rossol et al., 2012b).

Another example describing a CaSR-mediated inflammatory process triggered by macrophages is orchitis, which can induce male infertility (Su et al., 2020). Rat testicular macrophages showed upregulation of CaSR after infection, associated with increased activation of the NLRP3 inflammasome and release of IL-1β. Here, an increase in cytosolic [Ca^2+^] was also considered causative (Su et al., 2020). Studies from our group showed that macrophages from adipose tissue of patients with obesity respond with markedly increased IL-1β release after calcium stimulation of CaSR. These results suggest that CaSR-mediated inflammasome-associated processes may contribute to chronic inflammation in this disease (Thrum et al., 2022).

## Local imbalance of calcium homeostasis and calciprotein particles

The maintenance of human calcium homeostasis is complex and is safeguarded by different and partly redundant mechanisms. Independent of cell-mediated processes, biophysical phenomena are important. In this context, serum proteins such as fetuin-A, which act as inhibitors of ectopic crystallization, play an important role (Schinke et al., 1996; Brylka and Jahnen-Dechent, 2013). The negative charge of the elongated β-sheet within the amino-terminal cystatin-like D1 domain of fetuin-A (or α2-Heremans Schmid glycoprotein) enables the binding of Ca^2+^ and phosphate ions (P_i_) from serum to form soluble amorphous particles called calciprotein particles (CPPs) (Heiss et al., 2003). Similar to lipid-transferring apolipoproteins, fetuin-A serves as a vehicle for minerals and prevents the precipitation of crystals (Tiong et al., 2022). CPPs can mature sequentially and are categorized based on their size and structure. The spontaneous particle formation of Ca^2+^ and P_i_ in conjunction with monomeric fetuin-A leads to the generation of calciprotein monomers (CPM) (Rochette et al., 2009; Heiss et al., 2010). These monomers can fuse to form polymeric primary CPPs (CPP I) that contain amorphous calcium phosphate and can reach diameters of up to 100 nm (Smith et al., 2020). Secondary CPPs (CPP II) are crystalline complexes and exhibit hydroxyapatite. They are elongated ellipsoid shaped and reach a length between 100 and 250 nm (Smith et al., 2020; Kutikhin et al., 2021).

Under physiological conditions, the formation of CPPs in serum serves to balance fluctuations in mineral metabolism and is essential for maintaining calcium homeostasis in the body (Jahnen-Dechent et al., 2020). In a genetic mouse model, knockdown of the fetuin-A gene induces calcification of the myocardium and soft tissues (Schafer et al., 2003). Consequently, these protein-mineral complexes represent a dynamic component for stabilizing the local mineral balance and basically counteract pathological deposition of minerals (Schafer et al., 2003; Jahnen-Dechent et al., 2020; Smith et al., 2020). Recent studies by Koeppert and colleagues suggest that CPM degradation proceeds *via* the kidney. The contained fetuin-A is presumably reabsorbed by epithelial cells of the proximal renal tubules and excretion of calcium phosphate proceeds *via* urine (Koeppert et al., 2021). Previously, liver sinusoidal endothelial cells were found to be critical for the clearance of CPP I whereas kupffer cells eliminated secondary CPPs in addition to primary ones (Köppert et al., 2018).

In a pathological context, elevated levels of CPPs have been described particularly in association with vascular calcification (Yamada et al., 2014). CPPs have been identified in the tunica media of calcified arteries (Wu et al., 2020). Also, in patients with hypertension (Pruijm et al., 2017) and coronary atherosclerosis (Nakazato et al., 2019), increased serum CPP levels are detected. In patients with chronic kidney disease, high levels of CPPs are considered a causal factor for vascular calcification, which carries the risk of lethal cardiovascular complications (Hamano et al., 2010; Miura et al., 2018). Recently, however, it has also become clear that during aging, in parallel to an age-related decline in skeletal muscle mass, the level of circulating CPP increases (Yoshioka et al., 2022).

It has been described that cellular uptake of the particles can cause pro-inflammatory and cytotoxic consequences. Cells studied to date in this regard include vascular and valvular endothelial cells, vascular smooth muscle cells, adventitial fibroblasts, and interstitial cells of heart valves, as well as monocytes (Kutikhin et al., 2021).

The determination of CPP in serum or other biological fluids, its differentiation from membrane-associated particles, and its precise measurement despite its instability, are challenging tasks, as reviewed recently by Smith and colleagues (Smith et al., 2020). To date, the use of gel filtration methods (Miura et al., 2018) and the fluorescent bisphosphonate-based probe *OsteoSense* in conjunction with nanoparticle flow cytometry (Smith et al., 2017) are considered most adequate to examine the levels of CPPs in serum or other biological fluids.

## Calciprotein particles and calcium-sensing receptor-mediated macropinocytosis

Interestingly, experiments using different cell culture media demonstrated that CaSR-mediated activation of NLRP3 and IL-1β release in macrophages/monocytes are strictly dependent on the presence of phosphate in the media (Muñoz-Planillo et al., 2013; Jäger et al., 2020). This observation strongly implied the formation of some sort of calcium phosphate complexes, salts or crystals. However, crystallization in biomimetic fluids can most likely be excluded due to strong crystallization-inhibiting mechanisms in serum. Accordingly, no crystals are detectable by light microscopy in the concentration range used (Jäger et al., 2020). Only transmission electron microscopy made it possible to detect nanoparticles in culture media after addition of 2.5 mM [Ca^2+^]_ex_ (Jäger et al., 2020). Those particles were found to contain calcium, phosphate, and fetuin-A, which identifies them as CPPs as described by Jahnen-Dechent and colleagues and other groups earlier (Jahnen-Dechent et al., 2020).

**FIGURE 1:**
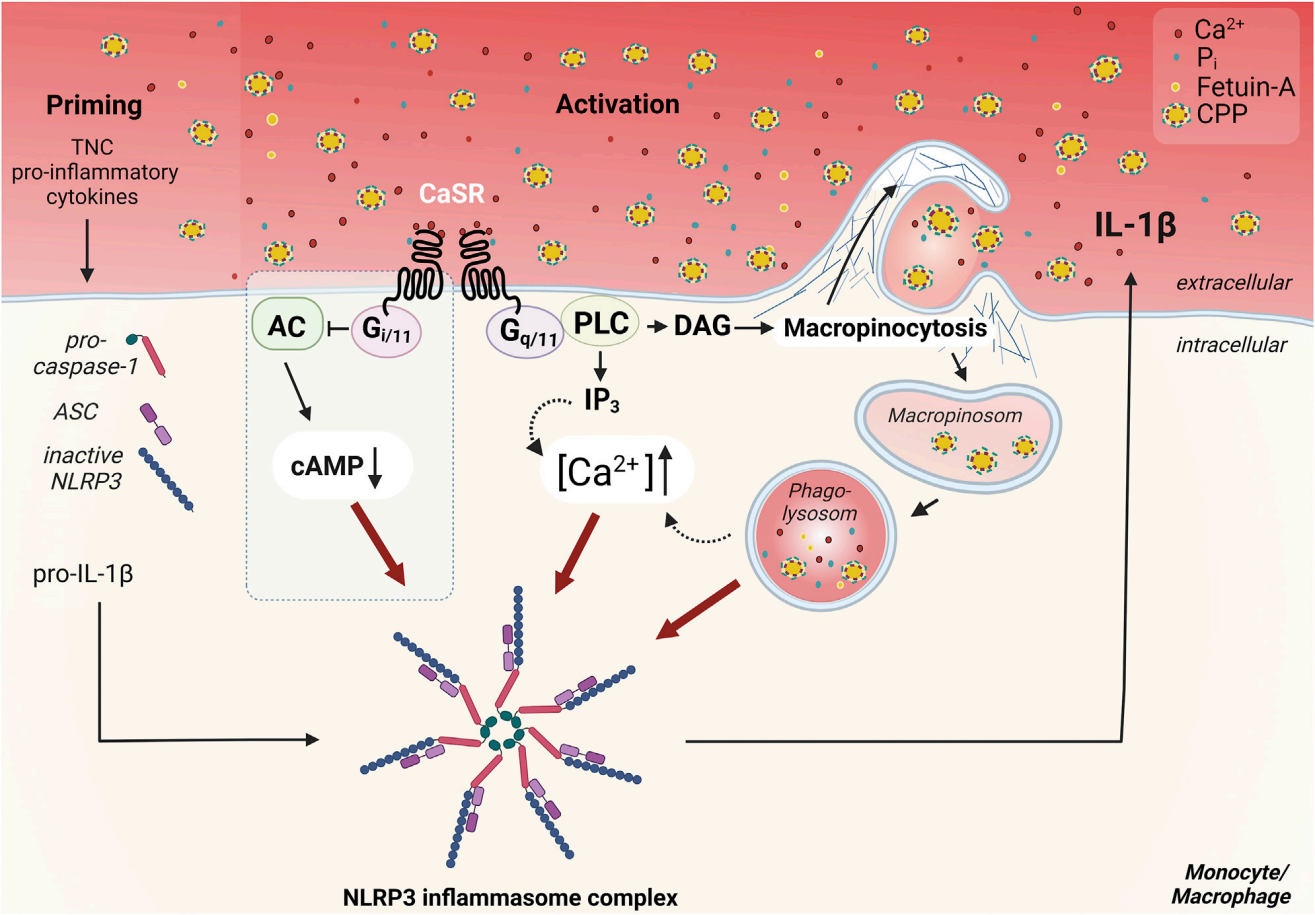
Overview of the CaSR dependent mechanism of Ca^2+^/CPP-induced inflammasome activation in monocytes/macrophages. CaSR-induced signaling pathways important for NLRP3 inflammasome activation, are induced by coupling to G protein dimers. Signal transduction *via* Gq/11 induces the activation of phospholipase C (PLC), which completes the hydrolysis of phosphatidylinositol 4,5-bisphosphate in inositol trisphosphate (IP3) and diacylglycerol (DAG). IP3 leads to release of Ca^2+^ from intracellular calcium stores. The resulting increase in intracellular Ca^2+^ concentration ([Ca^2+^]) triggers various signal transductions and presumably contributes to the activation of the NLRP3 inflammasome. CaSR-mediated Gi/o pathway activation, which has been described in macrophages (Lee et al., 2012) but not in monocytes (Rossol et al., 2012b), leads to inhibition of adenylate cyclase (AC) and reduction of cellular cAMP level. The downregulation of cAMP diminishes its inhibitory effect on the assembly of the inflammasome, therefore allowing for its activation (blue box) (Lee et al., 2012). Activation of CaSR also contributes to constitutive macropinocytosis in monocytes/macrophages and leads to accumulation of certain lipid mediators. These lead to actin polymerization *via* induction of specific GTPases such as Rac1/2, resulting in Arp2/3-dependent branching of the actin network. CaSR-mediated macropinocytosis allows the uptake of calciprotein particles (CPPs) from the extracellular space. These amorphous particles are formed *via* binding of Ca^2+^ and phosphate ions (Pi) by the serum protein fetuin-A. The uptake and internalization of these particles is pivotal for the activation of the inflammasome and induce an additional increase in cytosolic [Ca^2+^]. The priming necessary for the assembly of the inflammasome, which is required, for example, for the provision of pro-IL-1β, ASC, and pro-caspase-1, can be mediated by cytokines in the inflammatory context, or by endogenous toll-like receptor ligands like tenascin-C (TNC) in synovitic joints in rheumatoid arthritis. Figure modified according to (Jäger et al., 2020) and created with BioRender.com.

Such particles are unlikely to interact with the CaSR and trigger a receptor signal. However, myeloid cells like monocytes and macrophages engulf all content of the extra-cellular fluid by constitutive macropinocytosis, including CPPs. Therefore, the results of Canton and colleagues were of utmost importance (Canton et al., 2016) since they showed that constitutive macropinocytosis is crucially dependent on CaSR expression. This suggested that [Ca^2+^]_ex_-induced NLRP3 activation is mediated by macropinocytotic uptake of CPPs (see Figure 1). We were able to confirm this [Ca^2+^]_ex_- and CaSR-dependent uptake of CPPs and could show, that it triggers IL-1β release in low phosphate media (Jäger et al., 2020). Consequently, this can explain the phosphate dependency of the process, since phosphate is required during CPP generation, while it is dispensable in the cellular processes of CaSR-dependent macropinocytosis and subsequent NLRP3 assembly and activation. We were also able to further analyze the intra-cellular processing of the CPPs, which are transferred from macropinosomes to phagolysosomes and are digested by lysosomal hydrolases like cathepsin B (see Figure 1). Contrary to the effect of crystalline particles on lysosomes, no lysosomal leakage is triggered. Instead, lysosomal degradation of CPPs is critical for NLRP3 activation, since cathepsin inhibitors are able to inhibit IL-1β release (Jäger et al., 2020).

## Calcium-sensing receptor-triggered IL-1β release in rheumatoid arthritis

Rheumatoid arthritis (RA) is a chronic autoimmune disease characterized by a pathological autoimmune response which can precede clinical symptoms by years. In the context of an increased susceptibility due to the presence of RA associated HLA-DRB1 alleles, this immune response involves both T cells and B cells, and results in the generation of auto-antibodies against citrullinated peptides (ACPA). Those ACPA, in turn, are associated with a more severe course of the disease, and in particular, with a more rapid destruction of joints and bone matrix unless efficient treatment is initiated early.

**FIGURE 2:**
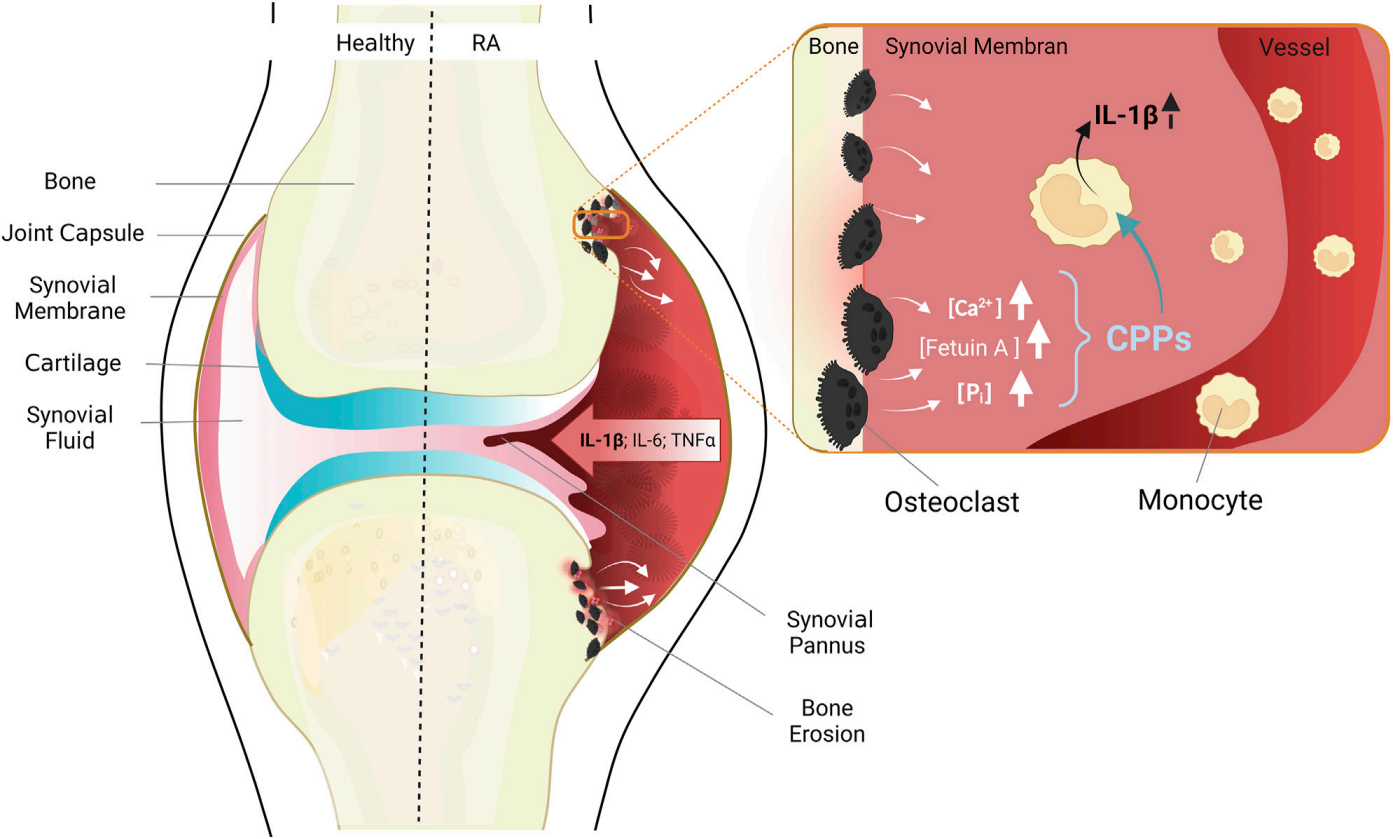
Anatomy of healthy joints (left) and pathological changes in RA (right). The healthy joint with joint capsule, synovial membrane consistent of a cell monolayer, cartilage and bone. RA leads to thickening of the synovial membrane due to immigrating immune cells, inflammatory cytokines are released, and cartilage and bone destroyed. Enlarged cutout: situation in areas of erosive bone resorption: Misdirected activation of osteoclasts leads to increased bone resorption, and release of high concentrations of the bone components calcium, phosphate, and fetuin-A. Monocytes migrate chemotactically from blood vessels into the synovial membrane, take up CPPs, and secrete pro-inflammatory cytokines, especially IL-1β. Figure modified according to (Jäger et al., 2020) and created with BioRender.com.

The earliest sign of such a bone destructive process is periarticular osteoporosis, which can be detected already at the earliest stages of disease, and which is therefore likely to have preceded them (Bøyesen et al., 2009; Güler-Yüksel et al., 2009; Hoff et al., 2009; Wevers-de Boer et al., 2015). Early metacarpal bone mineral density loss also predicts the pace of radiological joint damage later in the course of the disease, indicating that periarticular osteoporosis and erosive joint destruction are indeed closely linked (Bøyesen et al., 2009; Wevers-de Boer et al., 2015). In addition, clinical studies of individuals, which are positive for anti-CCP antibodies, but have no arthritis or clinical symptoms yet, have shown that lesions with subclinical inflammation in bone and bone marrow (Matthijssen et al., 2019) or early erosions (Brinck et al., 2018; Wouters et al., 2020), which are detectable only by magnetic resonance imaging (MRI), often precede development of arthritis. Those early changes in bone structure must be linked to resorption of bone matrix, and consequently, to local increases of the calcium and phosphate load which needs to be buffered and removed *via* the bloodstream (see Figure 2).

Already in early *in vitro* experiments with monoclonal antibodies blocking specific cytokines, it became clear, that cytokines produced by myeloid cells – like IL-1β, IL-6, and TNFα – play a pivotal role in the pathogenesis of RA (Brennan et al., 1989). Consequently, inhibitors of those cytokines were the first biological treatments for RA ever approved (Elliott et al., 1994; Bresnihan et al., 1998), thereby underlining the relevance of monocytes and macrophages for the chronic inflammation and bone destruction in this disease.

Monocytes of the peripheral blood have been shown to be crucially involved in the induction and maintenance of pathophysiological inflammatory processes of RA (Davignon et al., 2013). Our group has intensively investigated phenotype (Rossol et al., 2012a) and function (Meusch et al., 2013) of circulating monocytes in RA. We found increased intrinsic cytokine production, in particular of IL-1β, and this IL-1β release exerted clear pro-inflammatory and anti-apoptotic effects on monocytes in a paracrine and autocrine fashion *in vitro* (Meusch et al., 2013; Meusch et al., 2015). More recently, monocytes have received renewed interest due to the results of single cell RNA sequencing experiments on large numbers of RA patient samples, in which IL-1β producing monocytes were identified as a distinct cell population of pathogenetic relevance (Zhang F et al., 2019). IL-1β is a strong promoter of the inflammatory reaction and correlates with the extent of inflammation of the synovial membrane (Kinne et al., 2007). In combination with TNFα, which is also highly enriched in the synovial membrane of RA, it is a very potent stimulator of synovitis (Klimiuk et al., 2001).

In this context, the migration of monocytes from peripheral blood into synovial membranes (Lioté et al., 1996; Torsteinsdóttir et al., 1999) is of relevance. Once immigrated, the monocytes contribute to the perpetuation of the chronic immune response and inflammation in the rheumatoid synovium (see Figure 2). The inflammatory milieu within the joints in turn affects the differentiation of monocytes to osteoclasts, leading to increased bone resorption (Takayanagi, 2007; Nakashima and Takayanagi, 2008).

The described alterations of calcium homeostasis and the phenotypical and functional changes of circulating monocytes in RA lead us to hypothesize that the CaSR-dependent stimulation of macropinocytosis in RA monocytes contributes to initiation and perpetuation of the chronic inflammatory response in those joints due to the triggering of a cytokine cascade spearheaded by IL-1β, and followed by other myeloid cytokines (see Figure 2) (Rossol et al., 2012b; Jäger et al., 2020). This activation of monocytes – and possibly other immune cells present in the rheumatoid synovium – might then contribute to increased calcium concentrations in the intercellular space due to calcium release from activated cells, thereby fueling a vicious cycle.

Based on this hypothesis, we investigated peripheral blood monocytes from patients with RA and found them to respond with significantly higher IL-1β release to increased [Ca^2+^]_ex_, compared to healthy controls. Importantly, the stronger IL-1β response was related to disease activity, since patients with very early and active disease, who had not been seen by a rheumatologist or treated with steroids previously, had an even higher response compared to patients in remission. In the collagen II-induced arthritis mouse model, the link between [Ca^2+^]_ex_ and disease activity was confirmed, since the [Ca^2+^]_ex_-induced IL-1β response of mouse monocyte correlated closely with the arthritis score. Patients with other autoimmune arthritis like psoriatic arthritis or systemic lupus erythematodes, in contrast, did not differ in their [Ca^2+^]_ex_-induced IL-1β response from healthy controls.

The cause for this increased response in RA is likely related to CPP uptake, since monocytes from RA patients also show increased macropinocytotic uptake of CPPs compared to healthy controls. Simultaneously, we found increased expression of the CaSR on monocytes from RA patients. This might lead to stronger signaling and subsequently higher IL-1β release and in agreement with previous reports on increased CaSR expression in patients with RA, which then was associated with heightened cardiovascular morbidity and mortality (Paccou et al., 2014).

Two essential prerequisites for [Ca^2+^]_ex_-induced IL-1β release are the presence of sufficient concentrations of CPPs as well as a pro-inflammatory milieu contributing to TLR-mediated priming of monocytes. The former is certainly guaranteed in inflamed joints with bone erosion, since eroded bony matrix releases not only calcium and phosphate, but also high concentrations of fetuin-A, the most abundant non-collagenous protein in bone. The latter, the required TLR priming, is *in vivo* certainly not provided by LPS and TLR4, which are commonly used *in vitro*. There are, however, numerous endogenous TLR ligands, among them tenascin-C (TNC) which triggers TLR4 and can prime for [Ca^2+^]_ex_-induced IL-1β release (Midwood et al., 2009; Rossol et al., 2012b). Therefore, the required conditions for CaSR-mediated NLRP3 activation appear to be present in RA (see Figure 2).

When the local environment in arthritic joints was investigated more closely, we found increased calcium concentrations both in synovial fluid from RA patients as well as in arthritic mouse joints. In rheumatoid synovium from RA patients, the CaSR is overexpressed (Jäger et al., 2020). We propose, therefore, that this interplay between increased calcium concentrations, CaSR expression, and [Ca^2+^]_ex_-induced IL-1β release contributes to the perpetuation and possibly also to the initiation of the inflammatory disease process in RA joints.

## Summary and future outlook

The CaSR is expressed on myeloid immune cells and in particular on monocytes and macrophages. In an inflammatory setting, the receptor exerts stimulatory effects on monocytes and macrophages. *Vice versa*, expression and function of the receptors are also influenced by inflammatory stimuli and signals. The most dominant effector mechanism of CaSR stimulation in monocytes and macrophages is the assembly and activation of the NLRP3 inflammasome, subsequent caspase-1 activation, IL-1β release, and pyroptosis. This pro-inflammatory mechanism is strictly dependent on the presence of phosphate and fetuin-A in the extracellular fluid, since in addition to ligand binding to CaSR, macropinocytotic uptake of fetuin-A-based CPPs is strictly required.

This mechanism has been shown to be of relevance under various pathological conditions and in several diseases. In RA, it contributes to the chronic inflammatory disease process in arthritic joints, and is likely fueled by the generation of calcium protein particles during erosion of bone matrix. The pro-inflammatory effect is further amplified by an increased propensity of RA monocytes to hyper-react to extracellular calcium. More detailed knowledge about this pro-inflammatory mechanism will open up avenues to new therapeutic approaches and will facilitate the development of pharmacological therapeutics.
